# Eco-Friendly Method for Wood Aerogel Preparation with Efficient Catalytic Reduction of 4-Nitrophenol

**DOI:** 10.3390/gels9120978

**Published:** 2023-12-13

**Authors:** Qianqian Yu, Xiaohan Sun, Feng Liu, Zhaolin Yang, Shulei Wei, Chengyu Wang, Xin Li, Zechen He, Xiaodong Li, Yudong Li

**Affiliations:** 1 College of Chemistry and Bioengineering, Hechi University, Hechi 546300, China; 2022660001@hcnu.edu.cn (Q.Y.);; 2 Guangxi Key Laboratory of Sericulture Ecology and Applied Intelligent Technology, Hechi University, Hechi 546300, China; 3 Key Laboratory of Bio-Based Material Science and Technology of Ministry of Education, Northeast Forestry University, Harbin 150040, China; 18845890158@163.com (X.S.); wangcy@nefu.edu.cn (C.W.); 4 Infrastructure and Maintenance Section, Logistics Management Service, Hechi University, Hechi 546300, China; 5 Guangxi Collaborative Innovation Center of Modern Sericulture and Silk, Hechi University, Hechi 546300, China

**Keywords:** wood aerogel, deep eutectic solvents, chemical properties standardization, dye reduction, life cycle assessment, mulberry branch

## Abstract

The advancement of science and technology and the growth of industry have led to an escalating discharge of domestic sewage and industrial wastewater containing dyes. This surge in volume not only incurs higher costs but also exacerbates environmental burdens. However, the benefits of green and reusable catalytic reduction materials within dye processes are still uncertain. Herein, this study utilized the eco-friendly deep eutectic solvent method (DESM) and the chlorite-alkali method (CAM) to prepare a cellulose-composed wood aerogel derived from natural wood for 4-nitrophenol (4-NP) reduction. The life cycle assessment of wood aerogel preparative process showed that the wood aerogel prepared by the one-step DESM method had fewer environmental impacts. The CAM method was used innovatively to make uniform the chemical functional groups of different wood species and various wood maturities. Subsequently, palladium nanoparticles (Pd NPs) were anchored in the skeleton structure of the wood aerogel with the native chemical groups used as a reducing agent to replace external reducing agents, which reduced secondary pollution and prevented the agglomeration of nanoparticles. Results showed that the catalytic reduction efficiency of 4-NP can reach 99.8%, which shows promises for applications in wastewater treatment containing dyes. Moreover, investigation of the advantages of preparation methods of wood aerogel has important implications for helping researchers and producers choose suitable preparation strategies according to demand.

## 1. Introduction

Organic dye contributes to water pollution and is a notable threat to the ecosystem [1]. Various dye treatment methods, including coagulant precipitation [2], biodegradation [3], and dye adsorbent [4], have been used to remove or degrade dyes. However, these traditional methods have disadvantages, such as low separation or reduction efficiency, can contribute to pollution, and are time- and energy-intensive. Compared with these traditional methods, efficient catalysts are beneficial for reducing organic pollution [5,6], noble metals with nanostructures [7], and their excellent physicochemical properties have been brought into focus by the field of catalysis [8,9]. Among the various kinds of noble metals, palladium nanoparticles (Pd NPs) are highly active and particularly interesting [10,11,12]. However, Pd NPs have nanostructures that are weakly stabilized and are rarely reused because they aggregate easily [13,14], and recycling of the dispersed NPs in solutions is energy-consuming. Fortunately, the immobilization of Pd NPs on polymeric membranes with high porosity is a promising strategy for achieving excellent reusability and high efficiency. Feng et al. fabricated a microreactor with a polydopamine functionalized surface coated by Pd NPs; the polydopamine coating acted as an adhesive and a reducing agent which overcame the low reactivity of polytetrafluoroethylene [15,16]. However, most polymeric membranes load and immobilize Pd NPs via the addition of an external reducing agent [17,18], adhesion agent, and protecting ligand, contributing to their low chemical activity [19,20,21,22,23]. Biomass materials like cellulose and chitosan are used to support Pd NPs because of their high chemical activity, and because they can be widely sourced [24]. This can effectively reduce the use of additional reagents such as reducing agents and stabilizers. Nevertheless, the high throughput reduction of membrane reactors creates difficulties in practice, due to the low Pd-loaded content. Therefore, it was important to discover and design a material that has good wettability, excellent chemical activity, and a porous structure. Significantly, a cellulose-composed wood aerogel derived from natural wood for application in supporting Pd NPs is more beneficial for solving the above two problems because they are lightweight [25,26], hydrophilic, environmentally friendly, and have an anisotropic three-dimensional (3D) structure with a porous structure and excellent chemical activity [27,28]. The anisotropic structure (such as numerous long, partially aligned channels called lumens) and 3D high specific surface area (SSA) of wood aerogels are suitable for use as a separation material and a Pd NPs supporter for sewage treatment.

Usually, the strategy for preparing wood aerogels is top-down wood treatment technology [29], in which the chemical components of wood are removed in one-step or multi-step chemical treatments. Compared with natural wood, a wood aerogel with high SSA was a good candidate for reducing, stabilizing, and immobilizing Pd NPs, in order to open block channels and increase SSA. Among top-down wood treatment technologies, the traditional chlorite-alkali method (CAM) is a two-step method for removing the components of wood in which a sodium hypochlorite treatment is used to remove lignin and an alkali treatment is used to remove hemicellulose. The merit of CAM is that it standardizes the chemical properties of totally different species of wood.

Deep eutectic solvent (DES) has been extensively used in many fields such as bioactive and biomass refinery [30,31] because it has the characteristics of both ionic liquids and organic solvents, and possesses the advantages of inexpensive fabrication, insensitivity to water, and has techno-economically applicability [32,33]. Moreover, using DES as a chemical pretreatment strategy for wood substrates is very effective in that it endows the wood substrate with high porosity and activity for subsequent Pd NPs loading. The one-step DES method (DESM) requires less time, obtains by-products like lignin, and is easily recycled through distillation, indicating that DESM conforms to the concept of being environmentally sustainable. DESM has attracted considerable attention on account of its eco-friendly characteristics [34]. However, comprehensive studies and discussions on the use of DESM and CAM in the process of wood aerogel preparation are scarce. Furthermore, the downstream application potential of wood substrates might be hindered because of the lack of a systematic evaluation of the pretreatment process.

Herein, an eco-friendly method for wood aerogel preparation, the abovementioned DESM is proposed, and a life cycle assessment (LCA) was employed to estimate the environmental impacts (EIs) of two pretreated methods [35]. Furthermore, FTIR was used to detect different species and maturities of wood, which provided further proof of the CAM in standardizing the chemical properties of totally different wood species. This will help to guide decision-making regarding feedstock pretreatment in future research designs. Subsequently, the pretreated wood samples were decorated with Pd NPs via a hydrothermal method for wood reduction and the support of Pd NPs. The chemically active group of wood participated in independent Pd NPs reduction as a bio-reducing agent and stabilizing agent, and the holistic multi-porous skeleton of wood provided support for Pd NPs, which act like a polymeric membrane to solve problems related to agglomeration and recycling of Pd NPs. In addition, the structural effect of the pretreated wood combined with the high Pd NPs content was shown to enhance catalytic activity. Thus, the novel wood aerogel has the advantages of a low environmental impact to better address practical demands, and high sustainability for use in the treatment of dyed sewage. In particular, the in-depth study of the advantages of DESM and CAM for wood aerogels provides a guide for researchers and producers when choosing suitable pretreatment methods.

## 2. Results and Discussion

### 2.1. Morphological Characterization

**Figure 1:**
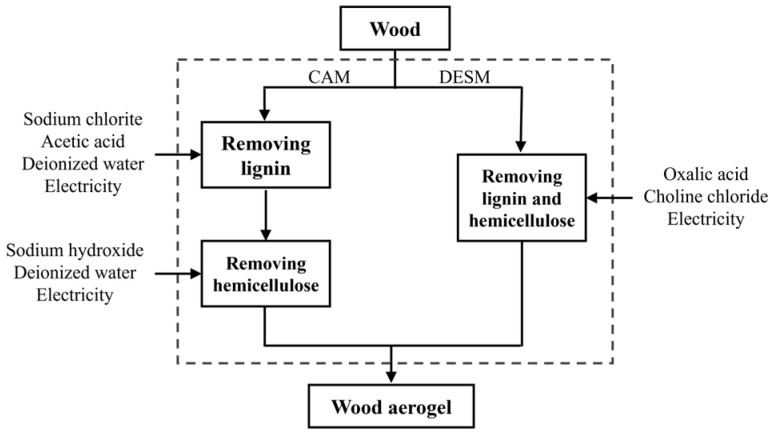
Flow chart of CAM and DESM.

Two independent projects of preparing the wood aerogel are shown in Figure 1. The route of CAM is divided into two main sectors, which are lignin removal via chlorite treatment and hemicellulose removal by alkali treatment. By comparison, the DESM preparation process is simpler, as lignin and hemicellulose are removed at the same time. To further investigate the morphology of the pretreated wood in CAM and DESM, a scanning electron microscope (SEM) was employed.

**Figure 2:**
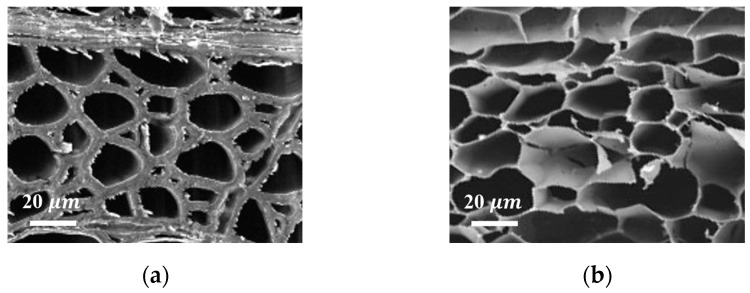
Cross-section of SEM images before and after DESM pretreatment: (**a**) NBS; and (**b**) DBS.

Natural balsa (NBS) was taken as an example to discuss the difference in the structure before and after the DESM pretreatment (DBS), and high-magnification SEM images of the cross-section of the NBS and the DBS were recorded (Figure 2a,b). There is no obvious change in the honeycomb-like porous structure, which indicated that DESM had no effect on the structure of NBS except the attenuation of the cell wall caused by partial delignification and the de-hemicellulose process in the DES. Additionally, the specific surface area (SSA) increased from 12.25 m^2^·g^−1^ of NBS to 82.32 m^2^·g^−1^ of DBS due to the removal compositions.

**Figure 3:**
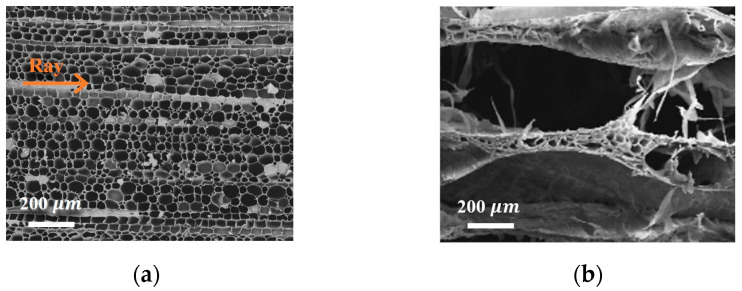
The broader view of SEM images after CAM pretreatment: (**a**) cross-section of DLBS; and (**b**) cross-section of DHBS.

Similarly, NBS was taken as an example to discuss the difference in structure after the CAM pretreatment. By comparing the low-magnification SEM images of the cross-section of the NBS with delignified (DLBS) (Figure 3a) and de-hemicellulose (DHBS) (Figure 3b), it is clear that, after removing the lignin and hemicellulose, the original honeycomb-like structure of DLBS was split in the direction of the wood ray (arrow direction in Figure 3a), and turned into the spring-like lamellar structure of DHBS (Figure 3b). The reason for the structural change was that the thin lamellas were cracked along the rays and stressed by ice crystals after the CAM treatment.

To sum up, DESM attenuated the cell wall but had no effect on the honeycomb-like structure of the NBS. By comparison, CAM had an effect on the original honeycomb-like structure and turned it into a spring-like lamellar structure, which indicates that the CAM removal components were more thorough. 

### 2.2. Chemical Functional Groups Characterization

To further distinguish the chemical properties of pretreated wood in CAM from DESM, Fourier transform infrared spectroscopy (FTIR) was employed. The molecular structure of the components of the wood and details are presented in Table S1. Specific and common peaks of wood appeared between 2000–550 cm^−1^, which is the fingerprint area of the wood species representing the moieties of cellulose, hemicellulose, and lignin. On the basis of the diverse cell structure and chemical composition among the different wood species, the woods were classified as softwood or hardwood. To further investigate the impact of CAM on the chemical properties of wood, Mongolian pine (softwood, labeled MP), balsa, beech, and mulberry (hardwoods, labeled BS, BC, M, respectively) were selected for FTIR analysis. Specifically, mature mulberry and mulberry branches (MB) were chosen to explore the variations in chemical properties associated with tree maturity. FTIR was conducted on natural woods (labeled N before the wood species) as well as those treated with chlorite-treated wood (labeled DL before the wood species) and alkali-treated wood (labeled DH before wood species).

**Figure 4:**
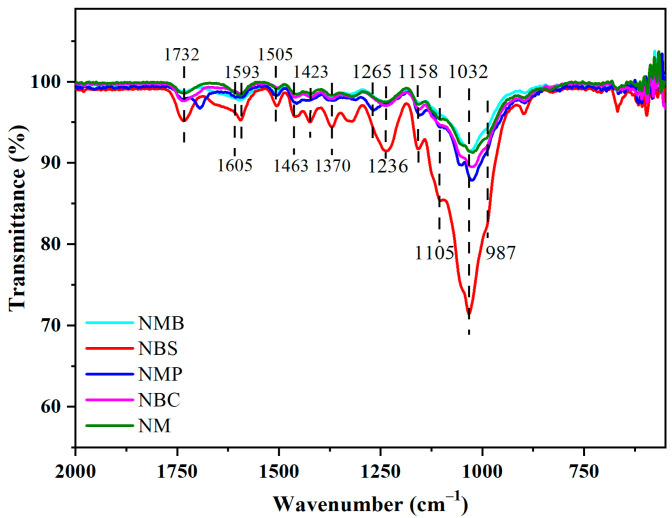
The FT-IR spectra of various natural woods.

**Figure 5:**
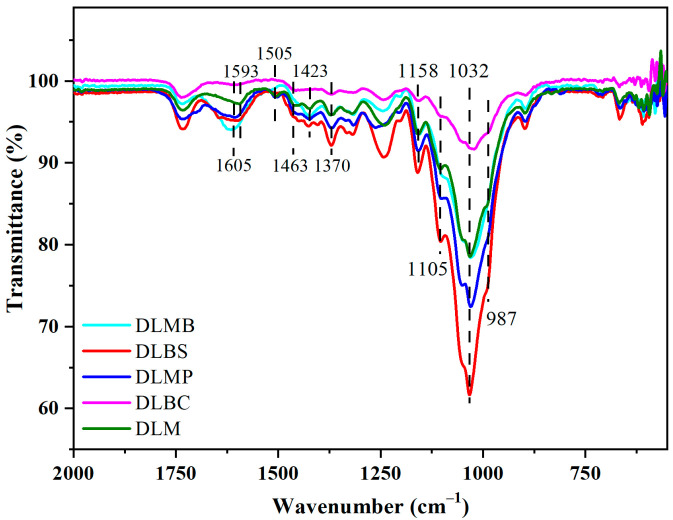
The FT-IR spectra of chlorite treated woods.

The FTIR spectra of NW among all the wood species shows common and special features with some differences in Figure 4. The most conspicuous, a discriminating band at 1605 cm^−1^ belonging to softwood, was sifted out from the band at 1593 cm^−1^ in other hardwoods, which corresponded to the conjugated ν C–O of the aromatic skeleton in lignin [36,37]. After pretreatment with chlorite, the peak was blue-shifted and attenuated, indicating that the molecular structure of lignin changed during the pretreatment with chlorite (Figure 5). 

**Figure 6:**
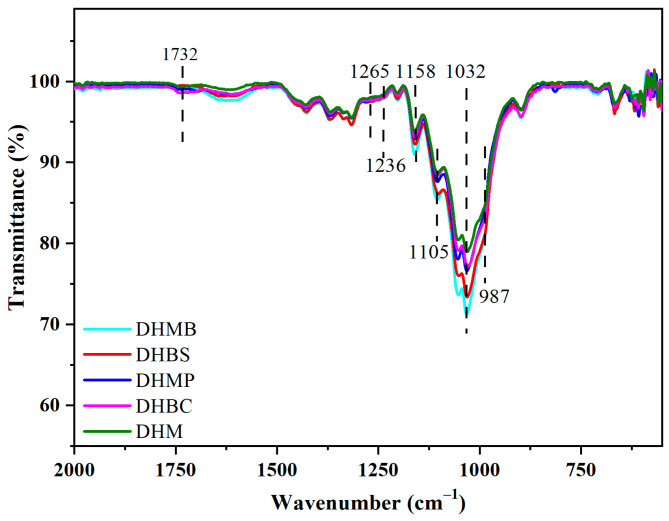
The FT-IR spectra of further alkali-treated woods.

As shown in Figure 6, the bands at 1463 cm^−1^ appointed to the formation vibration C–H in lignin and xylan, faded away after chlorite-alkali pretreatment. The bands at 1732 cm^−1^ appointed to ν C=O in xylan, and a discriminating band at 1265 cm^−1^ belonging to softwood, were sifted out from the band at 1236 cm^−1^ in other hardwoods, which corresponded to acetyl and hydroxyl of xylan. The bonds of xylan vanished after the alkali treatment. To sum up, CAM effectively removed most lignin and hemicellulose.

The bands at 1423 cm^−1^ represent δ C–H of cellulose, and the band at 1370 cm^−1^ is associated with the bending vibration C–H of cellulose. The abovementioned two band intensities did not change during the chlorite treatment process (Figure 5) and further alkali treatment (Figure 6), exhibiting that CAM hardly damaged the cellulose. Additionally, the shape of the bands at 1158–987 cm^−1^, representing ν C=O in cellulose and hemicellulose, tended to become homogeneous after chlorite treated and further alkali treatment. The chemical properties of hardwoods were unified, and the special features of the softwood disappeared (Figure 6), which indicated that CAM possesses the capacity to unify the chemical functional groups of various wood species. Moreover, mature mulberry and mulberry branches (MB) demonstrate slight distinctions due to differences in wood maturity (Figure 4). The chemical functional groups were unified via CAM (Figure 6), which indicated that CAM possesses the capacity to unify the chemical functional groups of various wood maturities.

Different geographical locations and climates result in plant diversity, and different species of wood are also composed of three main components, lignin, cellulose, and hemicellulose, but also have different chemical functional groups and molecular structures. Researchers or producers generally use local materials to reduce transportation costs and aid local economic development. This has also resulted in large differences in the chemical functional groups of similar products produced in different regions. Therefore, the chemical functional groups of different raw materials are unified by the CAM pretreatment, which can therefore achieve uniform products. Thus, this paper explores the advantages of CAM pretreatment from an innovative angle.

**Figure 7:**
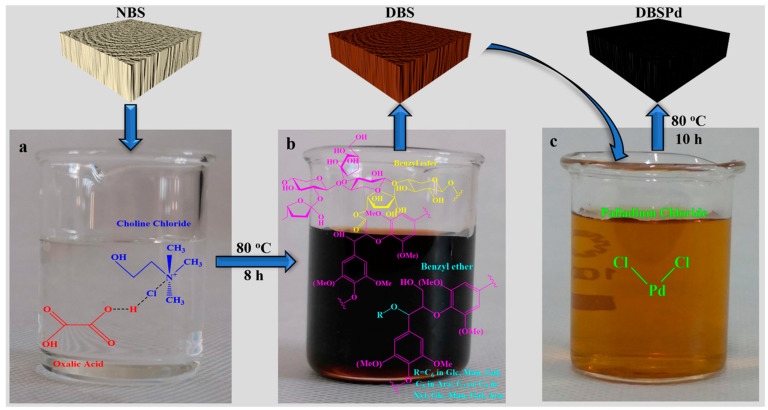
The flowchart of DESM treated NBS: (**a**) photo of DES, inset shows the DES composed of choline chloride and oxalic acid dihydrate; (**b**) photo of DES after treatment with NBS, inset shows the typical β-O-4 structure in lignin, the typical benzyl ester structures and benzyl ether structures in hemicellulose of the cleavage fragments; and (**c**) PdCl_2_ aqueous solution.

The ideal structure of DBS was obtained via an eco-friendly DESM using NBS with the removal of lignin and hemicellulose synchronously. Subsequently, a hydrothermal reaction was utilized to anchor Pd NPs on DBS and labeled as DBSPd (Figure 7). DES removes partial lignin and hemicellulose by breaking covalent bonds among these three main components of wood, and cleavage fragments (lignin and hemicellulose) are shown in Figure 7b. Lignin and hemicellulose are by-products of DESM, which is a benefit of DESM in addition to its environmental benefits.

**Figure 8:**
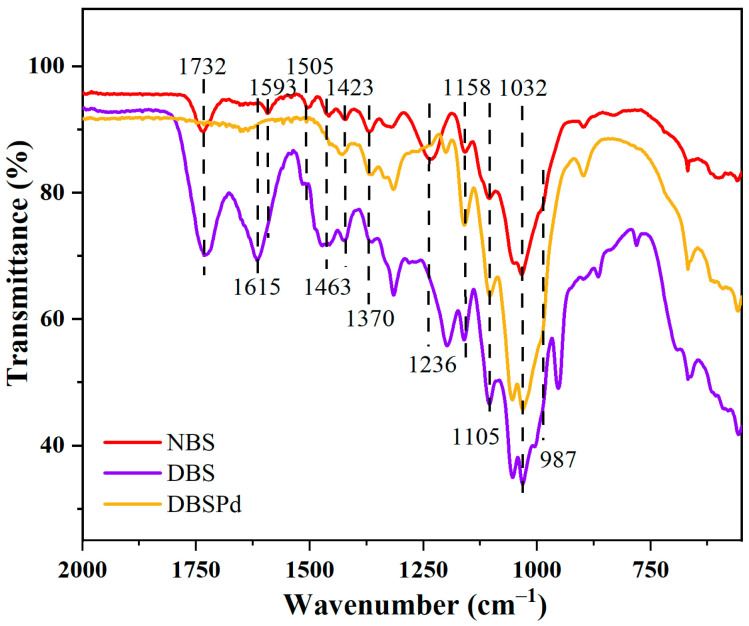
FT-IR spectra of NBS, DBS, and DBSPd.

Above mentioned partial components remove, which was further confirmed by FT-IR analysis (Figure 8). The partial removal of lignin can be inferred from the disappearance of characteristic peaks of lignin after DESM pretreatment, which located at 1505 cm^−1^ and 1463 cm^−1^. It was indicating that the content of lignin in wood was decreased. Besides, it could deduce from the vanish of xylan characteristic peaks at 1236 cm^−1^ of DBS that the significant amounts of hemicellulose were removed. After DESM pretreatment, the lignin-related peak at 1593 cm^−1^ was shift to 1615 cm^−1^ and increased, which was due to break bands could increase groups of the DBS [38]. Moreover, above mentioned enhanced peak (1615 cm^−1^) almost disappeared in FT-IR spectra of DBSPd, which verified that DBS reduced Pd^2+^ to Pd NP at the moment of absenting reductant. Overall, the lignin-related peak was shifted and increased after DESM pretreatment in stark contrast to the shifted and attenuated after CAM pretreatment, which is consistent with the conclusion that CAM removal components were more thoroughly obtained by SEM.

On the basis of the two independent wood pretreatment routes shown in Figure 1, LCA was employed to quantitively analyze the EIs of the CAM and DESM pretreatment processes. ISO 14040 and 14044 were used. The LCA process confirmed the goal and scope of the process, a life cycle inventory and an assessment. Finally, the results and discussion were followed by an uncertainty analysis. The details of LCA process are presented in Section 4.2. 

**Figure 9:**
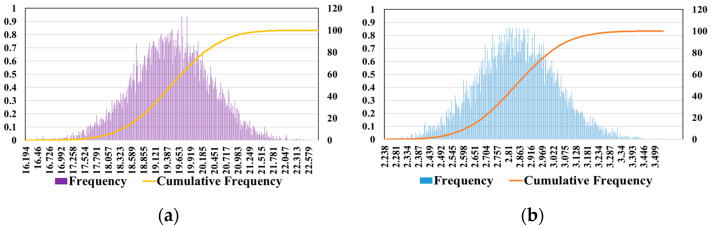
Frequency and cumulative frequency determined using Monte Carlo of GWP with: (**a**) CAM; and (**b**) DESM.

The uncertainty analysis of the results and discussion employed the Monte Carlo method, which described various random phenomena in the process. It is especially suitable for solving difficult and even impossible problems, and has an important position in the evaluation of uncertainty propagation. Therefore, a Monte Carlo simulation with ten thousand random numbers was used to consider the uncertainty propagation and deviation of materials and electricity depletion and to calculate the total effect and confidence interval (Figure 9a,b). 

**Figure 10:**
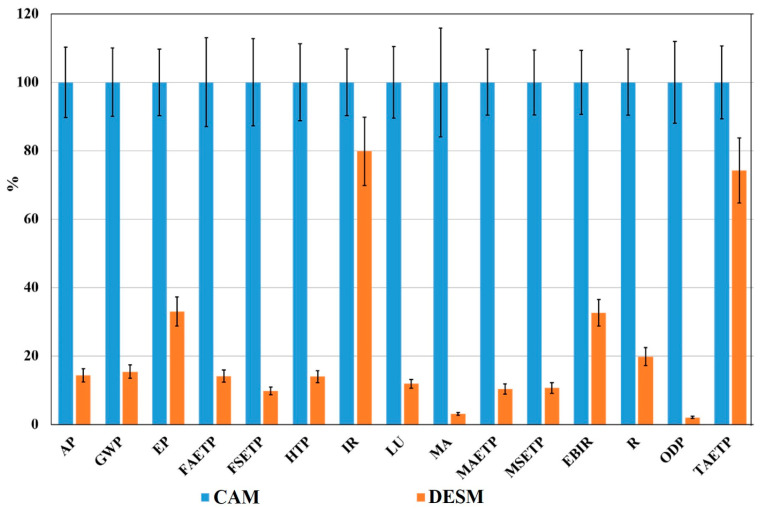
The total effect and confidence interval were calculated, and the relative indicator results of the respective project variants are presented.

In this article, CML 2001 was used to assess wood pretreatment processes in 14 categories, including: land use (LU), sediment ecotoxicity, the aquatic ecotoxicity of freshwater (FSET and FAET); the ecotoxicity of marine sediment, marine aquatic, and terrene (MSET, MAET, and TAET), potential eutrophication and acidification potential (EP, AP), climate change (GWP), human toxicity (HTP), ionizing radiation (IR), photochemical oxidation (EBIR), stratospheric ozone depletion (ODP), and resources (R). The results of the EIs in 14 categories of CAM and DESM are shown in Figure 10. It can be seen that the EIs of DESM were lower than those of CAM, which was attributed to higher material consumption (the list of input materials and energy of CAM and DESM are provided in Table 1). 

**Figure 11:**
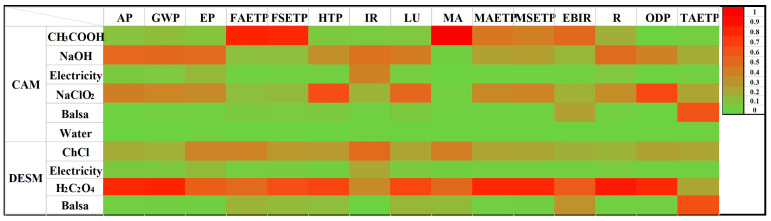
Sensitivity analysis results of LCA presented in color.

For DESM, the higher EIs contributed to climate change scores and human toxicity among 14 categories, which are clearly demonstrated in the sensitivity analysis results in color (Figure 11) and were caused by electricity consumption and preparation of the choline chloride. The phenomenon attributed to oxalic acid could be improved by changing hydrogen-bond donors (HBD) of DES to decrease EIs in DESM.

### 2.3. Applications in Dye Reduction

**Figure 12:**
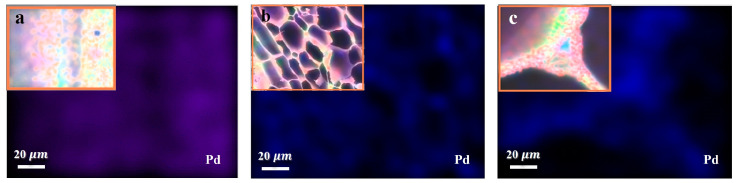
The results of EDS of: (**a**) longitudinal section; (**b**) cross-section; and (**c**) cell wall of the DBSPd.

The unique combination of the chemical components, and abundant and active functional groups of wood resulted in an even distribution of Pd NPs. The EDS images show that the internal and external surface of DBSPd evenly distributed Pd NPs without agglomeration (Figure 12). Additionally, the EDS for Pd NPs loading weight percentage was about 6.14% (containing C, O, Zr, and Pd) (Table S2).

**Figure 13:**
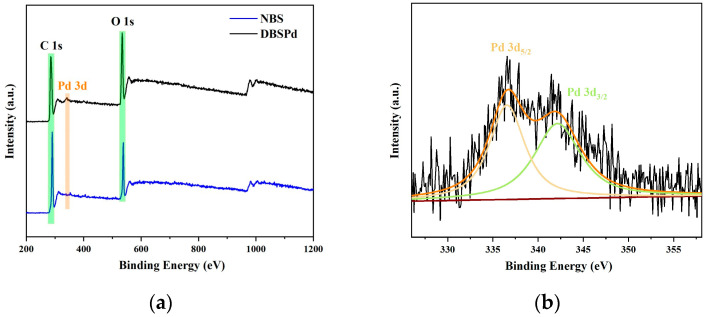
(**a**) XPS spectra of NBS and DBSPd; and (**b**) Pd 3d of DBSPd.

To further examine the chemical compositions and oxidation states of the Pd NPs of DBSPd, the XPS spectra of NBS and DBSPd are presented in Figure 13a. The peaks at 285, 341, and 532 eV corresponded to C 1s, Pd 3d, and O 1s, which indicate the Pd NPs loaded in DBS. In addition, the high-resolution XPS spectrum of the Pd 3d, respectively, showed double peaks at 335 and 342 eV (Figure 13b), and are attributed to Pd 3d5/2 and 3d3/2 [39]. These results show that Pd was successfully restored without any extra reducing agent.

**Figure 14:**
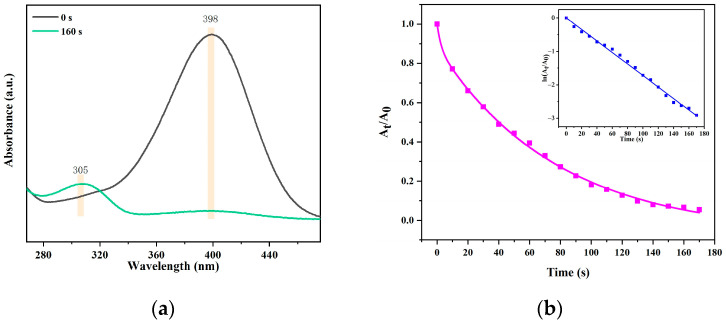
(**a**) The 4-NP UV-vis spectral results, when before reduction and after reduction, (**b**) plot of A_t_/A_0_ versus time for 4-NP reduction, and ln(A_t_/A_0_) versus time for 4-NP reduction presented in the inset.

The DBSPd application in 4-NP reduction was further evaluated. 4-aminophenol (4-AP) is widely used in the manufacturing of articles for daily use (e.g., analgesic, photographic developer) as a multipurpose raw material. Wood possesses adsorbability to dyes, but little could be ignored compared to the catalytic reduction part due to the small dosage [40]. In order to highlight the dye adsorption of NBS, the NBS was soaked in a methylene blue solution. As Figure S1 shows, the NBS turns blue, indicating adsorption for the dye but no catalytic reduction. The 4-NP reduction dynamic measurement was conducted using DBSPd with 20 mg·L^−1^ concentration. The 4-NP Ultraviolet characteristic band (398 nm) disappeared and a new absorption band of 4-AP (305 nm) was generated (Figure 14a). 

Furthermore, the 4-NP was reduced to 4-AP within 160 s with a reduction efficiency of 99.8%, and the plot of A_t_/A_0_ vs time is demonstrated in the pseudo-first-order kinetics in Figure 14b. In addition, the rate constant was counted for DBSPd (0.023 min^−1^) from the linear plot of ln(A_t_/A_0_) vs time presented in the inset [41]. Compared with other 4-NP reduction material in the previous work depicted in Table S3, DBSPd exhibits preferable properties of catalytic reduction. Above all, DBSPd exhibited excellent 4-NP reduction ability. It can not only treat hazardous pollution but also reduce product use in manufacturing, which is both environmentally friendly and helps to ensure chemical sustainability. Additionally, the structure of the DBSPd is loaded with Pd NPs, which reduce 4-NP. The 4-NP molecules are diffused on the structure of DBSPd and an electron transfer process occurs from NaBH_4_ to 4-AP when water containing 4-NP arrives at the center of the reduction. The high catalytic reduction efficiency of 4-NP is promising for applications in wastewater treatment containing dyes.

## 3. Conclusions

In summary, a cellulose-composed wood aerogel was fabricated by an eco-friendly DESM, which was verified by the LCA results. The DESM method has fewer environmental impacts than the traditional CAM method in pretreating wood. CAM was able to make the chemical functional groups of different wood species and maturities uniform. DBSPd was used to reduce 4-NP as an example. It exhibited excellent catalytic activity in the organic dye reduction, and the conversion rate of 4-NP was 99.8%. The enhanced catalytic activity of DBSPd contributed to the even distribution of Pd NPs on the internal and external surface of DBSPd without agglomeration, due to the catalyst space and improved catalytic behavior of the single Pd NPs. Therefore, this method is of great significance in removing dye contaminants, promoting environmental protection, and realizing sustainable development. Moreover, the in-depth study of the advantages of pretreatment methods for wood aerogels has important implications for research and production.

## 4. Materials and Methods

### 4.1. Materials

Balsa (*O. pyramidale*) came from Yueke New Material Co., Ltd. in Shanghai, China. Beech (*Zelkova schneideriana*), Mulberry (*Morus alba* L.), and Mongolian pine (*Pinus sylvestnis var. mongolica* Litv.) were purchased from Linyi North Wood Products Co., Ltd. ( Shandong, China). Mulberry branches (*Morus alba* L.) were picked from Yizhou district of Guangxi Province. Supplies of sodium borohydride (NaBH_4_) and acetone were provided by Beilian Chemical Co., Ltd. (Tianjin, China) and Xilong Scientific Co., Ltd. (Guangdong China), respectively. Oxalic acid dihydrate, sodium chlorite (NaClO_2_), acetic acid, and p-nitrophenol (4-NP) were supplied by Aladdin Biochemical Technology Co., Ltd. (Shanghai, China). Macklin Biochemical Technology Co., Ltd. (Shanghai, China) provided the choline chloride (ChCl) and palladium chloride (PdCl_2_). Hydrochloric acid (HCl) and sodium hydroxide (NaOH) were supplied by Kaitong Chemical Reagent Co., Ltd. (Tianjin, China). Anhydrous ethanol was supplied by Guanghua Sci-Tech Co., Ltd. (Guangdong, China). The abovementioned chemicals were used without further purification.

### 4.2. Life Cycle Assessment

International Organization for Standardization (ISO) 14040 and 14044 were followed to confirm the goal and scope of the LCA, life cycle inventory, and assessment. The results and discussion of the LCA were followed by an uncertainty analysis. The details of LCA were presented in following.

#### 4.2.1. Aim of LCA

The purpose of the LCA was to appraise the environmental impacts of CAM and DESM in wood treatment processes. Two independent wood pretreatment routes are presented in Figure 1.

#### 4.2.2. Inventory of Life Cycle

The inventory of the wood pretreatment process (mass and energy) was mainly determined by laboratory experiments and theoretical modeling [42]. On the basis of the eco-invent 3.4 databases, one chemical with an LCA inventory (as the scenario) was established [43].

The EIs of industrially producing choline chloride have not been collected in the database. Thus, the production of choline chloride was predicted by the R computing language rooted in a neural network algorithm. The results showed that 1.02592 kg of raw materials were sufficient for preparing 1 kg of choline chloride, which contained 0.21535 kg of ethane, 0.32994 kg of ethanol, 0.25818 of hydrochloric acid, and 0.22245 kg of methylamine.

The compositive elements of the wood pretreatment were established. The energy inventory for the electricity needed for wood substrate production was developed using a bottom-up approach, which was modeled via open-source LCA software with eco-invent 3.4 database. Detailed material charges are shown in Table S1.

#### 4.2.3. LCA

In this article, CML 2001 was used to assess wood pretreatment processes in 14 categories, which contained LU, FSET, FAET, MSET, MAET, TAET, EP, AP, GWP, HTP, IR, EBIR, ODP, and R.

#### 4.2.4. Uncertainty Analysis

A Monte Carlo simulation with ten thousand random numbers was used for the uncertainty propagation and deflected materials and electricity depletion, and to calculate the total effect and confidence interval.

### 4.3. Material Preparation

#### 4.3.1. Preparation of DBS and DBSPd 

The NBS was proceeded by following specific steps. DESM was used for the fabrication of the DBS, ChCl was coupled with oxalic acid dihydrate (1:1) to fabricate the DBS. Then, the Pd NPs were located by hydrothermal synthesis. The DBS was soaked with aqueous PdCl_2_ at 80 °C for 10 h. The DBSPd was successful fabricated following a process of freeze-drying.

#### 4.3.2. Fabrication of Delignification Wood and De-Hemicellulose 

CAM preparation of the wood aerogel was achieved by selectively removing lignin and hemicellulose from the wood cell wall, respectively, and then freeze-drying. The specific steps were as follows:

(1) Preparation of delignification (DL) wood. On the basis of the diverse cell structure and chemical composition among the different wood species, MP, BS, BC, M, and MB were selected for delignification. The wood was placed in an aqueous solution of 2% sodium chlorite (NaClO_2_) buffered by acetic acid (pH = 4.6) and reacted at 100 °C for 6 h, the delignification of the wood was achieved and labeled as DL before the wood species;
(2) Preparation of de-hemicellulose (DH) wood. The abovementioned delignified wood was placed in an 8% NaOH solution, reacted at 80 °C for 8 h, washed with deionized water after cooling to room temperature, and labeled as DH before the wood species.

### 4.4. Catalytic Behavioral Test

The catalytic behavioral assessment of DBSPd employed a model reaction of 4-NP and NaBH_4_ couple. The 4-NP concentrations were tested by UVa, and the 4-NP reduction efficiency was calculated as follows:

reduction efficiency (%) = 100 (C_0_ − C)/C_0_ (1)

where C_0_ and C are, respectively, the concentrations of the 4-NP solution before and after reduction.

A kinetic study of DBSPd (0.0075 g) in the system of 4-NP coupled with NaBH_4_ was performed on a quartz cuvette of 3 mL (4-NP (20 mg·L^−1^), NaBH_4_ (100 mg·L^−1^)). The ratio of the concentrations of 4-NP versus time, and the rate constants, were calculated as follows:

ln(A_t_/A_0_) = kt (2)

where k and t are the rate constant times, respectively, the values of A_0_ and A_t_ corresponded to the characteristic absorbance intensity of 4-NP at 0 and t, respectively. 

### 4.5. Characterizations

The surface morphologies of the wood slices were characterized with a scanning electron microscope (SEM, JSM-7500F, Tokyo, Japan). The SSA of NBS and DBS were determined using the Brunauer–Emmett–Teller method (BET, JW-BK132F, Beijing,China). An energy dispersive spectrometer (EDS, Bruker, Bremen, Germany) was used to map elements such as C, O, and Pd of the DBSPd. To determine the surface composition of the natural woods before and after DESM pretreatment of BS, and before and after CAM pretreatment of MP, BS, BC, M, and MB, samples were cut into small pieces and determined using Fourier transform infrared spectroscopy (FT-IR, frontier, Beijing, China). The surface composition of NBS and DBSPd was determined using X-ray photoelectron spectroscopy (XPS, K-Alpha, Waltham, MA, USA). The 4-NP reduction tests were confirmed using a UV−vis spectrometer (Cary 100, Melbourne, Australian).

## Figures and Tables

**Table 1:** List of inputs per functional unit for the baseline scenarios of the aforementioned two wood pretreatment processes.

Input	Amount	
	CAM	DESM
NBS	1.96 kg	1.86 kg
NaClO_2_	8.73 kg	/
Acetic acid	0.9813 kg	/
NaOH	10.4672 kg	/
Deionized water	124.2980 kg	/
Oxalic acid	/	4.4072 kg
ChCl	/	0.9762 kg
Electricity for heat	7.6880 kWh	4.9600 kWh

## Data Availability

The datasets analyzed or generated during the study have not been archived.

## Supplementary Materials

The following supporting information can be downloaded at: https://www.mdpi.com/article/10.3390/gels9120978/s1, Table S1: List of results of FTIR; Table S2: EDS results of DBSPd; Table S3: Comparison of DBSPd with other published materials for catalytic reduction of 4-Nitrophenol [20,44,45]. Figure S1: The photo of NBS after soak in a methylene blue solution.
