# Supplementary material for: Eco-Friendly Method for Wood Aerogel Preparation with Efficient Catalytic Reduction of 4-Nitrophenol
Source: Gels. 2023 Dec 13;9(12):978. doi: 10.3390/gels9120978 (PMC10743170; doi:10.3390/gels9120978)
Supplement: Supplementary file 1 [file gels-09-00978-s001.zip › gels-2671145-supplementary.pdf]

Supporting Information (SI)

Eco-friendly Method for Wood Aerogel Preparation with Efficient Catalytic Reduction of 4-Nitrophenol

Qianqian Yu [1,2,†], Xiaohan Sun [3,†], Feng Liu [1,2], Zhaolin Yang [3], Shulei Wei [1], Chengyu Wang [3], Xin Li [4], Zechen He [4], Xiaodong Li [1,2,5,*] and Yudong Li [3,*]

[1] College of Chemistry and Bioengineering, Hechi University, Hechi 546300, China; 2022660001@hcnu.edu.cn (Q.Y.); liufeng0517@163.com (F.L.); 17807893551@163.com (S.W.)

[2] Guangxi Key Laboratory of Sericulture Ecology and Applied Intelligent Technology, Hechi University, Hechi 546300, China

[3] Key Laboratory of Bio-Based Material Science and Technology of Ministry of Education, Northeast Forestry University, Harbin 150040, China; 18845890158@163.com (X.S.); 18845112698@163.com (Z.Y.); wangcy@nefu.edu.cn (C.W.)

[4] Infrastructure and Maintenance Section, Logistics Management Service, Hechi University, Hechi 546300, China; 2022660002@hcnu.edu.cn (X.L.); hezechen1994@sina.com (Z.H.)

[5] Guangxi Collaborative Innovation Center of Modern Sericulture and Silk, Hechi University, Hechi 546300, China

* Correspondence: lxdong_627@163.com (X.L.); ydli@nefu.edu.cn (Y.L.)

† These authors contributed equally to this work.

Table S1. List of results of FTIR

Chemical functional groups	Wavenumber (cm^{-1})
conjugated ν C–O of aromatic skeletal in lignin of softwood	1605
conjugated ν C–O of aromatic skeletal in lignin of hardwood	1593
formation vibration C–H in lignin and xylan	1463
ν C=O in xylan	1732
acetyl and hydroxyl of xylan of softwood	1265
acetyl and hydroxyl of xylan of hardwood	1236
δ C–H of cellulose	1423
bending vibration C–H of cellulose	1370
ν C=O in cellulose and hemicellulose	1158-987

Table S2. EDS results of DBSPd

Elements	weight percentage (%)	atomic percentage (%)
C	44.65	56.28
O	43.96	41.60
Zr	3.81	0.63
Pd	6.14	0.87

Table S3. Comparison of DBSPd with other published materials for catalytic reduction of 4-

Nitrophenol

Materials	k (min^{-1})	Time consuming (min)	4-Nitrophenol ($mg·L^{-1}$)
DBSPd wood aerogel (this work)	2.300×10^{-2}	3	$2.0×10^1$
PVDF@PDA@Au membrane [1]	4.784×10^{-3}	8	$1.0×10^2$
Au–PDA–PVDF membrane [2]	4.784×10^{-3}	5	$5.3×10^1$
Ag/PAN fibrous network [3]	3.600×10^{-2}	70	$2.5×10^1$

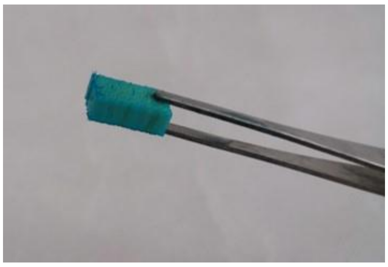

Figure S1. The photo of NBS after soak in a methylene blue solution

Reference

[1] J. Wang, Z. Wu, T. Li, J. Ye, L. Shen, Z. She, F. Liu, Catalytic PVDF membrane for continuous reduction and separation of p-nitrophenol and methylene blue in emulsified oil solution, Chemical Engineering Journal 334 (2018) 579-586.

[2] Z. Wu, H. Lin, Y. Wang, X. Yu, J. Li, Z. Xiong, Y. Wang, Y. Huang, T. Chen, F. Liu, Enhanced catalytic degradation of 4-NP using a superhydrophilic PVDF membrane decorated with Au nanoparticles, Rsc Advances 6 (2016) 62302-62309.

[3] S. Gao, Z. Zhang, K. Liu, B. Dong, Direct evidence of plasmonic enhancement on catalytic reduction of 4-nitrophenol over silver nanoparticles supported on flexible fibrous networks, Applied Catalysis B-Environmental 188 (2016) 245-252.
